# Thermodynamic and structural insights into the repurposing of drugs that bind to SARS-CoV-2 main protease

**DOI:** 10.1039/d1me00124h

**Published:** 2021-11-18

**Authors:** Shunzhou Wan, Agastya P. Bhati, Alexander D. Wade, Dario Alfè, Peter V. Coveney

**Affiliations:** Centre for Computational Science, Department of Chemistry, University College London UK p.v.coveney@ucl.ac.uk; Department of Earth Sciences, London Centre for Nanotechnology and Thomas Young Centre at University College London, University College London UK; Dipartimento di Fisica Ettore Pancini, Università di Napoli Federico II Italy; Institute for Informatics, Faculty of Science, University of Amsterdam The Netherlands

## Abstract

Although researchers have been working tirelessly since the COVID-19 outbreak, so far only three drugs – remdesivir, ronapreve and molnupiravir – have been approved for use in some countries which directly target the SARS-CoV-2 virus. Given the slow pace and substantial costs of new drug discovery and development, together with the urgency of the matter, repurposing of existing drugs for the ongoing disease is an attractive proposition. In a recent study, a high-throughput X-ray crystallographic screen was performed for a selection of drugs which have been approved or are in clinical trials. Thirty-seven compounds have been identified from drug libraries all of which bind to the SARS-CoV-2 main protease (3CL^pro^). In the current study, we use molecular dynamics simulation and an ensemble-based free energy approach, namely, enhanced sampling of molecular dynamics with approximation of continuum solvent (ESMACS), to investigate a subset of the aforementioned compounds. The drugs studied here are highly diverse, interacting with different binding sites and/or subsites of 3CL^pro^. The predicted free energies are compared with experimental results wherever they are available and they are found to be in excellent agreement. Our study also provides detailed energetic insights into the nature of the associated drug–protein binding, in turn shedding light on the design and discovery of potential drugs.

## Abstract 2

Drug repurposing is a tangible strategy for developing antiviral drugs in an emergency situation like the current COVID-19 pandemic.

## Graphical abstract

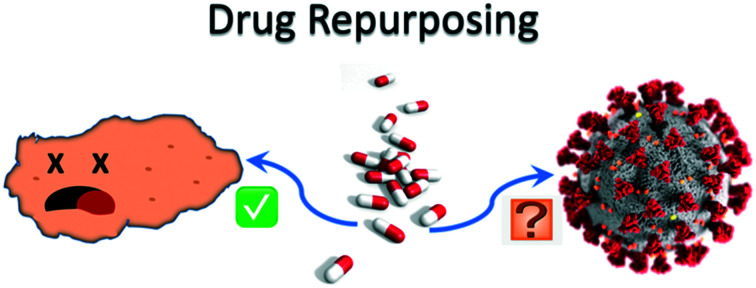

**Design, System, Application**

The COVID-19 pandemic has led to a rush to repurpose existing drugs which can treat the disease or arrest the spread of the virus. Drug repurposing can speed up the traditional process of drug discovery because the drugs have already been proven to be safe in humans. In the current study, we use molecular dynamics simulation and an ensemble-based free energy approach to investigate the interactions of a set of existing drugs with the main protease of the SARS-CoV-2 virus. The drug–residue interaction profile elucidates the amino acids crucial to the drug binding while the detailed energetic insights into the nature of binding shed light on possible new routes to future rational drug design.

## Introduction

From the start of the COVID-19 fight, scientists have been scrambling to find drugs that can treat the disease and perhaps even arrest the spread of the virus. Although many medications have been clinically tested for COVID-19 treatment, there are only three drugs – remdesivir, ronapreve and molnupiravir – which directly target the SARS-CoV-2 virus and have been approved or authorized for emergency use. Although working in entirely different ways, remdesivir and molnupiravir are broad-spectrum antiviral drugs targeting the RNA-dependent RNA polymerase (RdRp) of viruses. Ronapreve is an antibody cocktail containing two virus-neutralising antibodies, designed to target the spike protein of the coronavirus and to stop it attaching to the human angiotensin-converting enzyme 2 (ACE2). Studies have shown that remdesivir may only provide a modest benefit to patients with little to no effect on hospitalized patients with COVID-19.^1^ As a combination of two antibodies, ronapreve needs to be administered either by injection or infusion as quickly as possible after the first symptoms of illness. Molnupiravir has been approved by the UK medicines regulator for use in Covid-19 patients, although concerns remain on its mutagenic potential in human cells.

In general, small-molecule drugs have some obvious advantages over biologics, including their oral bioavailability, pharmacological activity, stability, permeability, *etc.* Relentless research efforts^2,3^ have led to some progress to find novel small-molecule drugs, or to repurpose existing small-molecule drugs for the treatment of the ongoing COVID-19 disease. An exciting global collaboration, called the COVID Moonshot, has come together for the discovery of new, urgent drug treatment for COVID-19.^4^ Despite this encouraging development, the discovery and development of new drugs are still associated with a slow pace and substantial costs. Repurposing of existing drugs for the ongoing disease therefore represents an attractive proposition as the safety of these drugs have been demonstrated in clinical trials and clinical applications.

The COVID-19 drug repurposing research can be grouped into four categories: clinical trials, *in vivo* cell experiments, *in vitro* protein-binding experiments, and *in silico* computational studies. As of 23 August 2021, there are 1822 registered COVID-19 clinical studies listed at https://clinicaltrials.gov/ct2/covid_view which have at least one drug intervention. There is a total of 615 drugs in these trials, many of which were initially developed for other diseases. *In vivo* and *in vitro* studies have uncovered some interesting drugs but caution must be exercised as the apparent antiviral activities are frequently caused by the drugs interrupting fundamental cellular processes rather than killing the virus or preventing its entry and duplication.^5^*In silico* computational research efforts are productive for the structures and interactions of the key proteins, especially at the earliest stages when no structures have yet been reported from experimental studies. Despite an unprecedented number of studies having been published in the last one and half years on computer-aided drug discovery,^6^ only one drug has arisen from computational studies^7^ – baricitinib – which has been approved for emergency use to treat COVID-19, in combination with remdesivir. Baricitinib is a repurposed medication: it is a kinase inhibitor originally designed to treat rheumatoid arthritis. As a broad-spectrum antiviral medication, remdesivir was originally developed to treat hepatitis C, and subsequently investigated for Ebola. Clinical trial has shown that the combination of baricitinib and remdesivir reduces the recovery time for hospitalized COVID-19 patients, especially for those requiring oxygen or ventilation.^8^ But neither alone, nor in combination, has proved to be a curative treatment for the disease.^8^ The four groups of repurposing studies are not independent. Computational studies, for example, require routine validation from experiments and clinics.

**Fig. 1:**
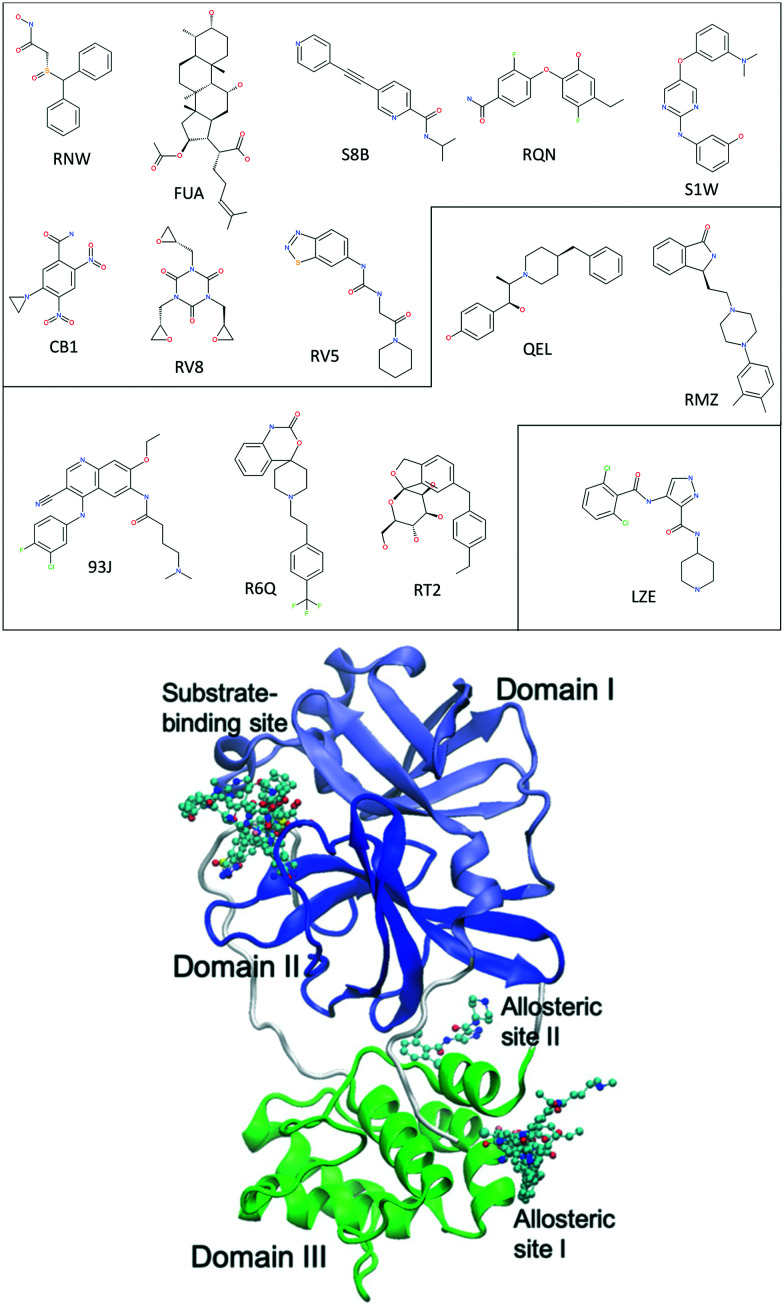
Chemical structures of 14 repurposing drugs to SARS-CoV-2 main protease. The drugs are grouped according to the sites they bind at: the substrate-binding site, the allosteric site I, and the allosteric site II (see Table 1). The drugs are also shown in chemical representation bound to 3CL^pro^ (shown in cartoon) at the three binding sites. The three domains (I, II and III) of 3CL^pro^ are shown in light blue, blue and green, respectively, along with some loops and links (white).

The main protease of SARS-CoV-2, 3CL^pro^ or M^pro^, is a key enzyme of the coronaviruses. It plays a pivotal role in processing the polyproteins that are translated from the viral RNA.^9^ The enzyme has become an attractive drug target because inhibiting its activity would block viral replication. X-ray structures have identified 22 different sites to which small molecules or molecular fragments can bind.^10^ However, most of the fragments do not show any antiviral activity. The most interesting small molecules in these x-ray structures are those screened from drug libraries in a recent study.^11^ A high-throughput X-ray crystallographic screening has been performed for two repurposing drug libraries against 3CL^pro^. The libraries contain 5953 drugs which have been approved or are in clinical trials, from which thirty-seven drugs have been identified which bind to 3CL^pro^. The effective concentrations of the drugs have been measured in a cell-based assay, at which SARS-CoV-2 infectious particles are reduced by 50% (EC50) (Table 1). Some of these drugs are considered to have antiviral activities, which show ≥100-fold reduction in infectious particles and have a much higher cytotoxic concentration than EC50 values.^11^ The X-ray structures of these drugs show that they bind at the substrate-binding site or one of the two allosteric sites identified^11^ (Fig. 1).

**Table 1:** Compounds bind with 3CL^pro^ noncovalently at the substrate-binding site and the two allosteric sites

Hit	Compound name	PDBe^a^	Tested in antiviral assay	pdb	EC50^c^ (uM)
Substrate-binding site					
#1	Adrafinil	RNW	+	7ANS	—
#11	Fusidic acid	FUA	+	7A1U	—
#18	LSN-2463359	S8B	−	7AWU	—
#20	MUT056399	RQN	+	7AP6	38.24
#27	SEN1269	S1W	−	7AVD	—
#34	Tretazicar	CB1	+	7AK4	—
#35	Triglycidyl isocyanurate^b^	RV8	+	7AQJ	30.02
#36	UNC-2327	RV5	+	7AQE	—
Allosteric site I					
#15	Ifenprodil	QEL	+	7AQI	46.86
#22	PD-168568	RMZ	+	7AMJ	—
#23	Pelitinib	93J	+	7AXM	1.25
#26	RS-102895	R6Q	+	7ABU	19.8
#32	Tofogliflozin	RT2	+	7APH	—
Allosteric site II					
#3	AT7519	LZE	+	7AGA	25.16

a PDBe ligand code.

b The drug binds to 3CL^pro^ covalently and non-covalently. The non-covalent binding mode is used in the current study.

c EC50 values from literature.^10^ “—” means no antiviral activity detected, or no cell assay performed.

**Fig. 2:**
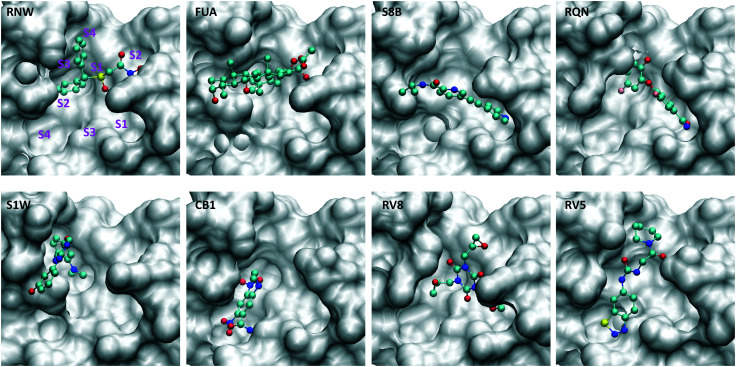
Drugs bind at different subsites within the substrate-binding site, which are labelled for one of the drugs (RNW). The conformational changes of the substrate-binding site are induced upon bindings of different drugs. The structures are generated from PDB IDs 7ANS, 7A1U, 7AWU, 7AP6, 7AVD, 7AK4, 7AQJ and 7AQE (Table 1).

In the current study, we use molecular dynamics simulation and an ensemble-based free energy approach, namely, enhanced sampling of molecular dynamics with approximation of continuum solvent (ESMACS),^12,13^ to investigate a subset of the aforementioned drugs. For the drug–protein complexes studied here, the binding sites of the protein are well structured, and the binding modes are resolved from crystallography experiments. It is likely that a reasonable prediction can be achieved, although the relatively large size of the substrate-binding site (Fig. 2) may pose a challenge for the conformational sampling and hence the convergence of the free energy predictions. In addition, per-residue free energy decomposition^14^ and close contacts between drugs and the protein have been performed to elucidate amino acids crucial to the binding and to understand the differential binding modes of drugs.

## Methods

Fourteen compounds were selected, all of which have X-ray structures available (Table 1) and non-covalently bind at one of the three binding sites: the substrate-binding site, and the allosteric sites I and II (Fig. 1). Two sets of ESMACS simulations were performed as follows: set-1 using individual X-ray structures for each of the compounds (see the PDB codes in Table 1), and set-2 using the same protein structure for all of the compounds. Set-2 simulation were performed because individual X-ray structures were not commonly available for all compounds in most drug development projects. In such projects, only one or limited number of protein structure(s) are available, to which the compounds are docked. Different initial structures have been shown to have a significant effect on the predicted binding free energies, especially when the timescale of simulations is short.^15^ In set-2, the structure 7AQE was used as it had the highest resolution among the PDB structures listed in Table 1. The backbone atoms of binding site residues, defined as those within 3 Å of any drug for a given binding site, were used for alignment of different protein structures. The structure 7AQE had drug RV5 bound at the substrate-binding site. For all other drugs binding at the same site, RV5 was replaced by them. No adjustment was made to the residues at the substrate-binding site for these drugs except S1W. The drug S1W had an obvious clash with the side chain of MET49, which was adjusted to accommodate S1W. For drugs binding at the allosteric sites, RV5 was removed and each drug was inserted into its binding site after alignment of the corresponding protein structure. The orientation of the sidechain GLN256 in 7AQE was also adjusted to better accommodate drugs at allosteric site I. All crystallographic water molecules were retained unless they overlapped with the inserted drug in set-2 models.

Drug parameterizations were created using the general Amber force field 2 (GAFF2).^16^ All drugs were electrostatically neutral except FUA which has a net charge of −1*e*. The AM1-BCC partial charges were assigned using the Antechamber component of the AmberTools package.^16^ The Amber ff14SB force field was used for the protein, and TIP3P for water molecules. The protonation states of the protein residues were assigned using the reduce module of AmberTools. All systems were solvated in orthorhombic water boxes with a minimum extension from the protein of 14 Å.

The binding affinity calculator (BAC)^17^ software tool was used to perform ESMACS studies. We employed an ESMACS protocol, which consisted of performing 25 replicas for a total of 10 ns production runs each. The molecular dynamics simulations were conducted using the package NAMD 2.14 (ref. 18) for each of the molecular systems studied. The protocol generates precise and reliable free energy predictions, and has been adequately validated for a diverse set of protein systems.^13,19–23^ The systems were minimized with all heavy protein atoms restrained at their initial positions, with restraining force constants related to their β-factors in X-ray structures. Initial velocities were then generated independently for each replica from a Maxwell–Boltzmann distribution at 50 K. The systems were virtually heated to 300 K over 60 ps with the NVT ensemble, followed by 2 ns equilibration with the NPT ensemble, during which the restraints on heavy atoms were gradually removed. Finally, 10 ns production simulations were executed with snapshots derived for analysis every 20 ps. A 2 fs time step was used for all MD simulations. NPT simulations were performed with pressure and temperature maintained at 1 bar and 300 K, respectively, during the equilibration and production runs. The simulations were performed on ARCHER2 (https://www.archer2.ac.uk/) and SuperMUC-NG (https://doku.lrz.de/display/PUBLIC/SuperMUC-NG).

## Results

The two sets of ESMACS simulations produce very similar free energy predictions for most of the drugs studied (Table 2). Only two drugs, RQN and FUA, have differences larger than 2 kcal mol^−1^ between the two predictions. Simulations in set-1 have been initiated from their individual x-ray structures, and are expected to be more reliable than those in set-2. Hereafter, we will focus on the results from set-1. The set-2 results will be discussed only to address the potential issues when a common protein structure is used for all of the compounds.

**Table 2:** Comparison of the predicted binding free energies (kcal mol^−1^) and the experimentally measured EC50 values (μM). The predictions were made from two sets of simulations, differing in the protein structures used. Bootstrapped errors, given to 67% confidence, are provided for the predicted energies. The drugs are ordered according to the predicted free energies from set-1 simulations

Ligand	Δ*G*^set-1^_ESMACS_	Δ*G*^set-2^_ESMACS_	EC50^a^
Substrate-binding site			
RQN	−22.16 ± 0.35	−15.98 ± 0.64	38.24
CB1	−21.19 ± 0.48	−21.09 ± 0.52	+
RV8	−20.70 ± 0.86	−21.29 ± 0.75	30.02
S1W	−20.69 ± 0.30	−18.77 ± 0.90	−
FUA	−18.07 ± 0.61	−12.98 ± 0.94	+
RV5	−17.77 ± 0.91	−19.07 ± 0.49	+
RNW	−13.20 ± 0.44	−13.57 ± 0.35	+
S8B	−12.38 ± 0.26	−11.69 ± 0.39	−
Allosteric site I			
93J	−19.86 ± 0.18	−19.78 ± 0.15	1.25
RT2	−18.36 ± 0.43	−18.59 ± 0.40	+
R6Q	−15.33 ± 0.46	−14.93 ± 0.41	19.8
RMZ	−13.55 ± 0.39	−14.05 ± 0.45	+
QEL	−12.18 ± 0.48	−11.78 ± 0.53	46.86
Allosteric site II			
LZE	−18.36 ± 0.32	−19.60 ± 0.30	25.16

a EC50 data from literature.^10^ “+”: cell assay performed but no antiviral activity detected at the highest concentration (100 μM) tested; “−”: no cell assay performed.

### Binding free energy ranking

For the drugs at the substrate-binding site, cell assays detect two drugs with antiviral activity, RQN and RV8. From simulations, RQN has the most favourable binding free energy, while RV8 is also one of the drugs with the most negative binding free energies. In cell assay, RV8 has slightly higher antiviral activity than RQN, as indicated by a lower EC50 concentration. ESMACS calculations observe a reversed order for their binding affinities. It should be noted that X-ray structures reveal both covalent and noncovalent binding modes for RV8. The antiviral activity may be attributable to both of the binding modes. In ESMACS simulations, however, only the noncovalent binding mode is studied, which may contribute to the differences between the experiment observations and the simulations.

For the drugs at the allosteric site I, excellent agreement is obtained between the two sets of simulations. The simulations generate not only the same ranking, but the same binding free energies, within error bars, for all of the compounds binding at this site (Table 2). The calculated binding free energies also agree with the experimental measurements for a subset of these drugs for which EC50 values are detected. For LZE at the allosteric site II there are no other drugs to compare with at the same binding site, but its binding free energy is very favourable. This drug has antiviral activity observed in the cell assays.^11^

### Per-residue contributions

The drugs studied here are highly diverse, interacting with different binding sites (Fig. 1), and different subsites for these at the substrate-binding site (Fig. 2). The subsites are defined as the regions where the amino acid residues of the polypeptide substrate bind. 3CL^pro^, like many other proteases, has an extended substrate-binding site which is spacious for common small-molecule drugs. The drugs interact with different subsites; residues at the binding site are expected to contribute differently to the bindings of the drugs.

**Fig. 3:**
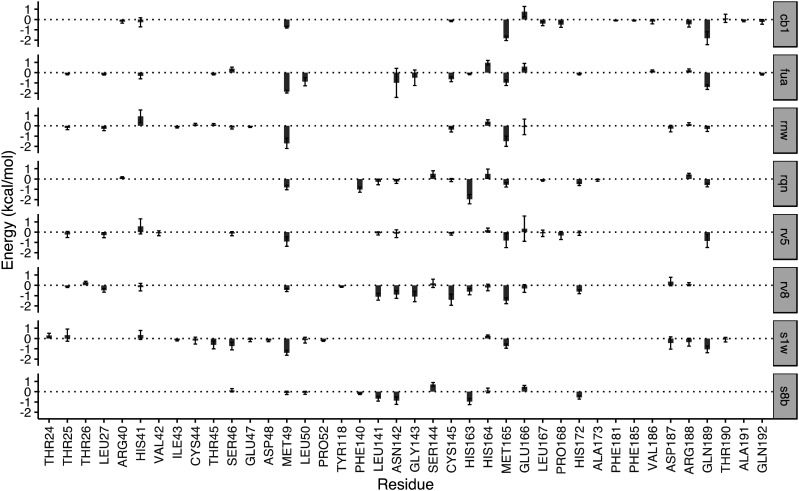
Decomposition of the binding free energy on a per-residue basis for the drugs in the substrate-binding site. The major contribution toward the binding free energy comes from a few clusters of residues, indicated by the negative residue–drug interaction energies. The error bars represent the variations of the energies from individual replicas. The residues with contributions between −0.1 and 0.1 kcal mol^−1^ are not shown for reasons of clarity.

To quantify the energetic contribution of each amino acid to the bindings, we have performed a per-residue decomposition analysis^14^ for the drugs at the substrate-binding site. It is observed that the residues contributing the most to the bindings are clustered with residue numbers between 25 and 27, 41 and 50, 140 and 145, 163 and 173, and 187 and 192 (Fig. 3). While most of these residues contribute to the bindings in a favourable way, some residues show unfavourable contributions to the binding energies. Although there is a total of 37 residues having a contribution of |Δ*G*| > 0.1 kcal mol^−1^, only one residue, MET49, is universally presented for all of the drugs at the substrate-binding site. Another residue, MET165, appears for 7 out of 8 drugs, and is merely missed for S8B with a contribution of −0.09 kcal mol^−1^. 23 of the residues have contributions (|Δ*G*| > 0.1 kcal mol^−1^) only for one or two drugs. The contributions from the same residue also vary significantly for the binding of different drugs. HIS163, for example, provides the most favourable contribution for one of the drugs, RQN, with an energy of −1.96 kcal mol^−1^. Its contributions for three other drugs, S1W, RV5 and CB1 are, however, negligible. It should be noted that the residues do not interact with the drugs independently. HIS163 contributes favourably for the binding of all eight drugs, while its immediately adjacent residue, HIS164, contributes unfavourably for 6 out of 8 drugs studied here.

In addition to the size and accommodation capacity of the subsites, the occurrence of mutations at the binding site should also be considered when designing new drugs or modifying existing drugs. Correlated mutation analysis (CMA) has revealed some key residues which contribute significantly to the protein stability.^24^ These residues are unlikely to mutate and thus can be considered as key anchoring residues for drug design. Some of these anchoring residues^24^ overlap with the ones which contribute significantly to the binding from the per-residue analyses. Residue G143, for example, is one of the anchoring residues identified from CMA study; it also contributes significantly to the binding energies of RV8 (−1.09 kcal mol^−1^) and FUA (−0.49 kcal mol^−1^). These interactions of drugs with the conserved residues are likely to be maintained within potential 3CL^pro^ variability, and hence reduce the probability of resistance emergence. On the other hand, drug resistance may be present when mutations occur in the residues important to the drug–protein interactions. Mutation of residue N142, for example, has been identified from a sequencing study.^25^ The residue contributes significantly to the binding of S8B, RV8 and FUA, with binding free energy contributions of −0.87, −0.90 and −0.97 kcal mol^−1^, respectively. The mutation may confer resistance against these drugs if the drug–residue interactions become less favourable. The interference of the neighbouring residues, however, would complicate the estimations of their individual contributions to drug binding affinities. This problem can be overcome by calculating the overall contributions instead from the neighbouring residues as a whole.

### Importance of the initial structures

Although most of the predicted binding free energies agree well from the two sets of simulations, obvious differences exist for two of the drugs: RQN and FUA (Table 2). The protein structure (PDB id: 7AQE) used in the set-2 simulations has high overall similarity to others, with a RMSD of main chain atoms less than 0.4 Å for all of the drugs except S1W. A close inspection of the binding site residues, however, reveals substantial differences. The differences can have a large impact on the propensity for and stability of drug binding. The orientation of MET49 in 7AQE, for example, reduces the size of the S3′ subsite (Fig. 2), making the binding site less able to accommodate drugs like QRN to which the fully appearance of S3′ subsite is crucial.

**Fig. 4:**
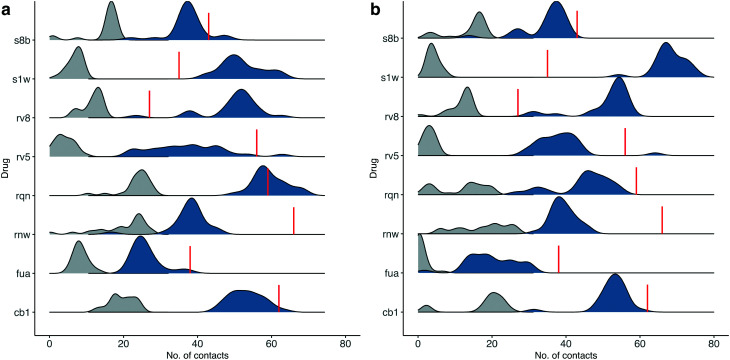
Number of contacts between drugs at the substrate-binding site and the protein for the simulations starting from their individual X-ray structures (a), or using the same protein structure (b). A contact is defined when the distance of heavy atoms between the drugs and the protein is less than 4 Å. The total contacts observed during the simulations are shown in blue, whereas a subset of these that overlap with those observed in the X-ray structures are shown in grey. The red lines indicate the numbers of contacts found in the X-ray structures.

**Fig. 5:**
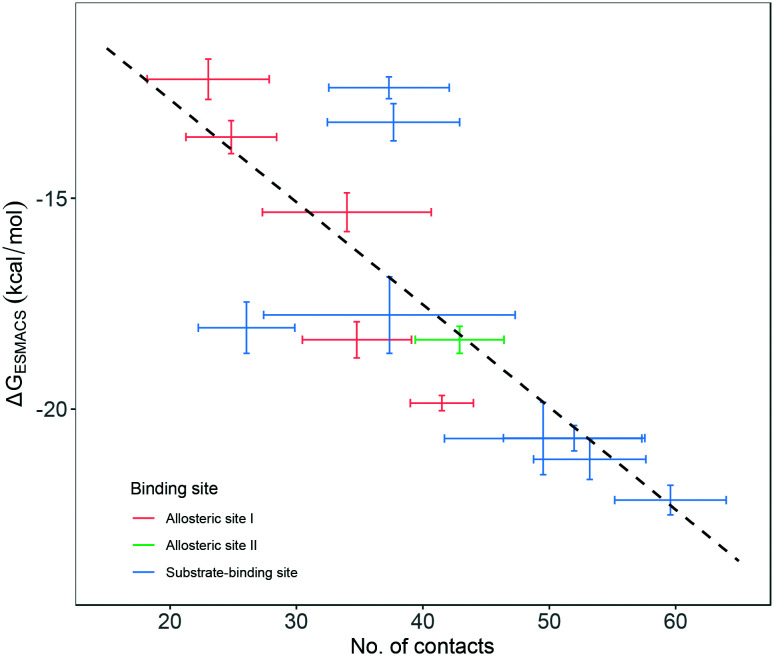
Correlation between the number of contacts and the binding free energies predicted from ESMACS approach. The error bars represent the standard deviations of the properties from ensemble simulations.

To check the stability of drug binding, the close contacts of the drugs with the protein are monitored (Fig. 4). A close contact is defined when the distance is less than 4 Å between two heavy atoms arising within drug and protein. The number of contacts is a good qualitative indicator of the predicted binding free energies.^26^ It has a good correlation with the binding free energies from the ESMACS approach, with a correlation coefficient of −0.78 (Fig. 5), indicating that more contacts lead to more negative free energies.

In set-1 simulations, 5 out of 8 drugs have less contacts during the simulations than those identified in the initial X-ray structures, and 2 drugs have more contacts. Only one drug, RQN, maintains roughly the same numbers of contacts as these in its X-ray structure. Most of the contacts from the X-ray structures are not maintained in the simulations. The lost contacts are largely compensated by other pairs of drug and protein atoms which are brought closer during the simulations. In set-2 simulations, all drugs except S1W have similar or less contacts comparing with those in set-1 simulations. This is not unexpected as conformational differences are usually induced when small molecules bind to a protein. A single protein structure is not optimal for the binding of every drug. Consequently, the results from set-2 simulations are less reliable than these from set-1. Some drugs may even drift away from the binding sites identified in the crystallography experiments. We are currently performing very long time scale MD simulations in which binding and unbinding events are investigated. The drug FUA in set-2, for example, is observed to either bind at different binding subsites or is completely unbound in the ensemble simulations, evidenced by its contact distribution extending to 0 (Fig. 4). This is the reason for its less favourable binding free energy predicted in set-2 simulations (Table 2).

## Conclusions

Using the ESMACS protocol, we have computed the free energies of a series of drugs binding to the SARS-CoV-2 main protease (3CL^pro^). The drugs are selected from an X-ray screening study, in which the three-dimensional structures have been determined for 3CL^pro^ complexed with a set of potentially repurposable drugs, and the antiviral activities been measured for some of them in a cell-based assay. The drugs studied here are highly diverse in their structural and chemical properties, and in their binding modes in the binding sites of the protein. For the drugs with antiviral activity detected in cell assay,^11^ the rankings of binding free energies from ESMACS approach are in excellent agreement with the experimentally determined drug potencies.

The per-residue energy decomposition provides some favourable features of the binding sites, which are important for the building of an energy-based pharmacophore model. The analysis suggests the amino acids crucial for the binding of drugs at the 3CL^pro^ binding sites. The combination of energy-based and structure-based pharmacophore models could provide an improved virtual screening for the initial selection of promising compounds.

Although the X-ray structures have a high degree of similarity for the particular set of drugs studied here, the difference in predicted binding free energies for the drugs RQN and FUA when using individual X-ray structures compared to using repurposed X-ray structures from similar protein ligand complexes, highlights the importance of the local conformations at the binding site. The orientations of some side chains need to be adjusted to better accommodate different drugs. While treating the protein as a rigid entity is still common for most docking studies, induced fit docking approaches have been attempted in which an ensemble of protein conformations is used. The conformations are collected from multiple experiments or more commonly are generated by MD simulations. The close contacts between the drugs and the proteins in the simulations differ significantly from those present in the x-ray structures. Our study provides detailed energetic insight into the nature of drug–protein binding, which may be used to shed light on the design and discovery of potential drugs.

## Data accessibility

The molecular models and force parameters are available at https://doi.org/10.23728/b2share.1c42a67a73e9424b8192ba65c81077e1

## Conflicts of interest

There are no conflicts to declare.

## Supplementary Material
